# Lunacy Reformation

**Published:** 1910-04-30

**Authors:** Forbes Winslow

**Affiliations:** 57 Devonshire Street, Portland Place, W.


### Medical Letter-Box.

### LUNACY REFORMATION.

*To the Editor of* The Hospital.

Sir,—My attention has been drawn to a leader in your issue of April 9. You have omitted the principal part of the question as to the nature of the resolutions I shall bring before a forthcoming meeting. The gist of this is that lunatic asylums are only for chronic cases or those who are dangerous to themselves or others. That there should be hospitals where acute cases, like puerperal mania, should be placed with a view of treating them, and so prevent the necessity of branding them with the stigma of lunacy, which is the only means we have at the present day. In these days of apathy we require someone who has the courage of his opinion and his convictions, and who is not afraid to act as he considers right and just in the cause of humanity. Judging from the number of letters I have received on the matter, I am more than encouraged by what I propose doing. We shall press our resolutions upon the Government, who are at present too much occupied in matters which do not in any way concern the welfare of the human race. The Veto and the Budget should, in my opinion, take a back seat in comparison with lunacy reformation. Your contemporary *John Bull* will, no doubt, be able to take care of himself in this matter. The "Eminent Surgeon," who went to the office to get his case made public, had every justification in doing so. All those who are connected with the case frankly admit that a wrong diagnosis was made, alcoholic delirium was diagnosed as General Paralysis of the Insane. This latter complaint does not, however, occur suddenly, and inasmuch as within a week of his admission to the asylum this "Eminent Surgeon" had performed successfully the operation for appendicitis, removal of the gasserian ganglion and cleft palate, it can hardly be said that a man suffering from incipient general paralysis of the insane could have done this. I have all the papers relating to his case in my possession, including copies of original certificates. It will be interesting to know the name of the doctor upon whom the assault was made. This is the first time I have heard of this. I was informed by him that he was struck by an attendant. At the present moment he is suffering from a serious bodily complaint, or possibly he would have communicated with you himself on this matter. This lunacy reformation is not the first agitation I have entered into. I am not ashamed of doing this, or, indeed, in any other matter where I consider that facts justify such an action. If, as you say, it "is in the estimation of the medical profession, and of the lunacy experts particularly, likely to be highly prejudicial to the real interests of the public," then I presume they would be content to allow the present unsatisfactory state of the law to remain in *status quo*, though condemned by authorities and others, by public inquiries and Royal Commissions. They have a right to their opinion, I have a right to mine, and I intend to act as I consider proper in this matter, and in the interests of those who are, unfortunately, unable to help themselves.

Faithfully yours,

Forbes Winslow.

57 Devonshire Street,
Portland Place, W.,
April 13, 1910.

[The only paragraph in this letter upon which we desire to offer any comment is that in which Dr. Winslow asks for the name of the doctor upon whom an assault was made. This we cannot give publicly; but we are quite prepared to communicate it privately, provided the consent of the medical man in question is first obtained. The fact we mentioned was got at first hand from this gentleman, who is now an officer in one of the Medical Services of the Crown.—Ed. The Hospital.]

